# Factors associated with the unsuccessful TB treatment outcomes in the northern regions of Namibia: a mixed methods study

**DOI:** 10.1186/s12879-023-08268-y

**Published:** 2023-05-22

**Authors:** Mondjila Amkongo, Honoré K. Mitonga, Anna Alfeus, Loide Ndelimona Ndapandula Shipingana, Tuwilika Keendjele, Hilja Eelu, Tunelago Nashihanga

**Affiliations:** 1 Department of Radiography, School of Allied Health Sciences, University of Namibia, P.O Box 3728, Windhoek, Namibia; 2 Public Health Department, School of Nursing and Public Health, University of Namibia, Windhoek, Namibia; 3 Department of Human, Biological & Translational Medical Sciences, School of Medicine, University of Namibia, Windhoek, Namibia

**Keywords:** Tuberculosis, DOTS, Treatment success rate, Namibia

## Abstract

**Background:**

Tuberculosis (TB) is among the leading causes of death globally. The disease has a huge burden in Namibia, with a case notification rate of at least 442 per 100,000. To date, Namibia is among the countries with the highest global TB burden, despite all efforts to reduce it. This study aimed to determine the factors associated with the unsuccessful treatment outcomes of the Directly Observed Therapy Short course (DOTS) programme in the Kunene and Oshana regions.

**Methods:**

The study utilised a mixed-methods explanatory-sequential design to collect data from all TB patient records and healthcare workers who work directly with the DOTS strategy for TB patients. The relationship between independent and dependent variables was analysed using multiple logistic regression analysis, while interviews were analysed using inductive thematic analysis.

**Results:**

The overall treatment success rates of the Kunene and Oshana regions throughout the review period were 50.6% and 49.4%, respectively. The logistic regression analyses showed that in the Kunene region, the type of DOT used (Community-based DOTS) (aOR = 0.356, 95% CI: 0.835–2.768, *p* = 0.006) was statistically significant with the unsuccessful treatment outcomes. While in the Oshana region, age groups 21–30 years old (aOR = 1.643, 95% CI = 1.005–2.686, *p* = 0.048), 31–40 years old (aOR = 1.725, 95% CI = 11.026–2.9, *p* = 0.040), 41–50 years old (aOR = 2.003, 95% CI = 1.155–3.476, *p* = 0.013) and 51–60 years old (aOR = 2.106, 95% CI = 1.228–3.612, *p* = 0.007) had statistically significant associations with the poor TB-TO. Inductive thematic analysis revealed that patients in the Kunene region were challenging to reach owing to their nomadic lifestyle and the vastness of the area, adversely affecting their ability to observe TB therapy directly. In the Oshana region, it was found that stigma and poor TB awareness among adult patients, as well as mixing anti-TB medication with alcohol and tobacco products among adult patients, was a prevalent issue affecting TB therapy.

**Conclusion:**

The study recommends that regional health directorates embark on rigorous community health education about TB treatment and risk factors and establish a robust patient observation and monitoring system to enhance inclusive access to all health services and ensure treatment adherence.

## Background

Driven mainly by the bacterium *Mycobacterium tuberculosis*, an acid-fast and rod-shaped bacillus, Tuberculosis (TB) is a poverty-driven disease and may cause affected individuals to experience economic hardship and vulnerability, Tuberculosis (TB) ranks among the top 10 causes of mortality globally and is the leading global cause of death from a single pathogen, causing approximately 1.3 million deaths globally [1, 2]. It may cause affected individuals to experience economic hardship, vulnerability, stigma, and discrimination [3]. Sub-Saharan Africa has the highest regional TB prevalence due to a high TB/HIV comorbidity. This remains a significant factor in the TB epidemic and associated mortality [4]. Moreover, the advent of Covid-19 has drastically reduced TB treatment outcomes (TB-TO), making it difficult to reach the set targets [5]. In this study, TB-TO was classified as either successful or unsuccessful. Successful TB-TO was comprised of the sum of those who are cured and those who completed treatment, while unsuccessful TB-TO is an aggregate of cases with the following status: treatment failure died, loss to follow-up or not evaluated.

In 2019, around 10 million people worldwide developed TB, with 1.2 million deaths among HIV-negative individuals, with an additional 208,000 deaths among those with HIV. Adults made up 88% of TB cases, while children accounted for the remaining 12% [6].

In the Central African Republic, the incidence of TB varied in 2019, with rates of 100–199 per 100,000 people in Cameroon and Chad, and rates of 300–499 per 100,000 people in the Democratic Republic of the Congo [3]. Tuberculosis incidence rates in the Central African Republic surpassed 500 per 100,000 people during the same period. In terms of TB mortality rates per 100,000 persons in 2019, Chad had around 22, Cameroon had 29, the Democratic Republic of the Congo had 49, and the Central African Republic had a stunning 98 [7]. Zimbabwe's TB incidence rate decreased from 242 per 100,000 in 2015 to 199 per 100,000 in 2019, which could be an indication of their transition out of the top 30 high TB-burden countries [8].

In Namibia, the disease has wreaked havoc, particularly among poverty-stricken communities. With a TB per capita incidence of 442 cases per 100 000, a case notification rate (CNR) of 487 per 100 000 and 60 people per 100 000 dying, Namibia was ranked fifth among countries with the highest global TB burden by the World Health Organization (WHO) [9, 10]. Since the Namibian government adopted the Directly Observed Treatment Short Course (DOTS) strategy to control TB in the 1990s, the disease has remained a serious health concern, despite it being both curable and preventable. By directly observing patients receiving treatment, the DOTS strategy aimed to increase adherence to TB treatment and reduce transmission [11].

The National Guidelines for Tuberculosis management necessitate various controls to tackle the disease. Diagnosing tuberculosis involves bacteriological methods, such as the Acid Fast Bacilli (AFB) smear test, and non-bacteriological methods like GeneXpert. In Namibia, the primary diagnostic tool is the sputum smear test, which is affordable, fast, and specific. However, for HIV-infected patients, additional tests may be needed. Chest radiography (CXR) serves as a secondary diagnostic method in Namibia, used when sputum smear tests are inconclusive or sputum is unavailable.

During the Third Medium Term Plan for Tuberculosis and Leprosy (MTP-III), global TB initiatives included a minimum target of successfully treating 90% of new TB cases [10]. High treatment success rates (TSRs) can be attained when nations improve health education, the follow-up to patients and socioeconomic support for patients at an increased risk of loss to follow-up. Moreover, nutritional supplementation, rapid treatment of adverse effects, healthcare workers’ commitment to TB interventions, patient adherence to treatment, and meaningful governmental health partnerships were key strategies to improve TSR [12]. Therefore, there is a need to increase efforts to ensure that Namibia exceeds the 90% target, as the nation has only successfully treated 88% of its new cases [10]. Namibia is comprised of 14 regions, of which two of the northern regions, Oshana and Kunene regions have consistently been among the top under-performers in TB mitigation, based on the TSR indicator during the Second Medium Term Plan for Tuberculosis and Leprosy (MTP-II). This means that the two regions never reached the 85% TSR target during the MTP-II. Additionally, the factors associated with the unsuccessful treatment outcomes in the two areas are unknown. This study aimed to identify, analyse and describe the factors related to the unsuccessful treatment outcomes in Oshana and Kunene regions. The health belief model was used to better understand the factors contributing to the DOTS program's low treatment success rates in the Kunene and Oshana regions. The study looked at how social and environmental factors affected TB treatment effectiveness. The goal was to identify and investigate the factors that contributed to poor tuberculosis treatment outcomes in these areas.

There is limited research on the factors affecting the underperformance of TB control programs in the worst-performing regions in Namibia. This study aims to address this knowledge gap by examining the factors associated with the DOTS program treatment success rates in Oshana and Kunene regions, providing valuable insights into intra-regional performance.

## Methods

### Study setting

Quantitative data were collected from the electronic TB register (ETR.net), in the Ministry of Health and Social Services’ headquarters, under the National Tuberculosis & Leprosy Programme in Windhoek. The qualitative data were collected from key informants (working closely with the TB control programme) from Kunene and Oshana. The majority of inhabitants in the Kunene region practice subsistence farming, livestock keeping, and small-scale agricultural cultivation. Tourism is also crucial to the local economy, with some inhabitants engaging in tourism-related activities. The Kunene region’s TB case notification rate varied over this study’s review period, having reduced from 294 to 225 per 100 000. The region houses three hospitals, three health centres and 24 primary health care clinics. The data were collected from the three district hospitals, namely: Opuwo, Outjo and Khorixas district hospitals.

Subsistence farming, with a focus on staple crops, goats, and cattle, is likewise the predominant activity in the Oshana region. Some residents work in the informal sector, while a substantial amount of inhabitants work in education or health care, among others. In the Oshana region, the TB notification rate was relatively higher than that of the Kunene region, having reduced from 550 to 405 per 100 000. This region has one hospital, five health centres and 12 primary health care clinics. The data were collected from the Oshakati Intermediate Hospital, Okatana and Ongwediva Health Centres as well as Ekamba and Uukwiyuushona clinics.

### Study design and periods

This study used a mixed-methods explanatory sequential design. The first phase used a quantitative retrospective design to collect data from the National Tuberculosis & Leprosy Programme’s (NTLP) ETR.net. This study abstracted data from all patient records captured in the ETR.net from 2016 to 2020 in Kunene and Oshana regions. Data were collected from these records using a six-item data abstraction tool and analysed to determine the factors associated with poor TSR in both regions. A pilot study was conducted on records from another area during the same review period to test the feasibility of the study. Records with missing treatment outcomes and those who transferred to the other areas were excluded from the study. Cases with missing values in other variables are accounted for by the use of listwise deletion.

The second phase employed a qualitative, phenomenological design to make sense of the meanings of key informants’ experiences regarding the factors identified in the initial phase. In this phase, individual voice-recorded structured key-informant interviews were conducted with TB field promoters and primary health care nurses working in six government health facilities in Kunene and Oshana regions, who were well embedded within the TB programme. Data were collected until saturation was achieved when themes or patterns started to repeat themselves, and no new insights emerged. One trained researcher conducted the interviews and analysed the data under supervision. To ensure dependability, audit trails were used during data analysis, including a description of the research methodology. Moreover, bracketing was used, meaning that the researchers stayed impartial while analysing the data and only reported what emerged from the participants' real experiences. This was achieved by combining non-proportional quota and purposive sampling, as the Kunene region has three health districts while Oshana has only one health district, although it had more health facilities than Kunene. After analysing and reviewing the quantitative data and results, the interview guide was developed.

### Population

The population for quantitative data collection were all the TB records captured in the ETR.net from all health facilities in Oshana and Kunene regions during the period under review.

The population for qualitative data collection were constituted of six healthcare workers directly involved with the TB DOTS programme in the Kunene region, as key informants for this project – this included four TB Field Promoters and three Primary Health Care (PHC) Nurses. The Oshana region’s population included eight healthcare workers directly involved with the TB DOTS programme, as key informants for this study– this included six TB Field Promoters and two PHC Nurses.

### Sample size determination and sampling technique

Since this study utilised an exploratory sequential mixed methods design, it required a combined form of quantitative and qualitative sampling methods. In the quantitative phase, all TB records captured in the ETR.net from Kunene and Oshana regions from 2016 to 2020 formed part of the sample. A combination of a non-proportional quota and purposive sampling was used in the qualitative methods section. The population was divided into groups (strata) and samples were obtained from each specified population sub-group to meet the desired specific numbers (quota). The region with one district was sampled by the numerous health facilities available, and then purposive sampling was used to select the participants who were interviewed. The inclusion criteria encompassed all TB records from Oshana and Kunene regions' health facilities and healthcare workers involved with the DOTS programme or TB patients in these regions. Exclusion criteria involved omitting cases with missing treatment outcomes, those who transferred to other regions, and health workers who did not work with the DOTS program.

### Data collection procedure

During the quantitative data collection, the researcher used a data abstraction tool, during working hours from the patient records in the ETR.net at the Directorate of Special Program’s NTLP, TB Division. After all the data was collected on the tool, it was migrated onto Microsoft Excel version 2016 for cleaning, and then imported to SPSS (version 26) for analysis.

Subsequently, qualitative data were collected from the selected key informants from the two regions over two months using structured interviews. The interviewees were contacted before setting an appointment at a time that does not inconvenience their work.

### Data processing and analysis

After completing the data collection phase, the data were migrated to Microsoft Excel 2016 and then SPSS version 26 for cleaning and analysis, respectively.

Subsequently, qualitative data were collected from the selected key informants from the two regions over two months using structured interviews.

Inferential statistics using logistic regression analysis with a 95% confidence interval (CI) determined the statistical significance of the relationship between the dependent variables (TB-TO) and the several socio-demographic and clinical characteristics and independent variables (such as age groups, sex, bacteriological results, HIV status, type of DOT). Pearson Chi-square.was also used to test the strength of association between the dependent variables and the identified predictor variables. The results were considered statistically significant if the *p*-value of ˂0.05 Qualitative data collection sought to explain the quantitative results. Once the second data collection phase was completed, the recorded qualitative data were transcribed, coded, and then themes and sub-themes were generated in line with inductive thematic analysis. This method was used to analyse the data and generate themes naturally occurring to avoid researcher bias.

### Ethical considerations

Permission to conduct the study was approved by the University of Namibia’s Human Research Ethics Committee (UREC) (Ref: OSHAC/594/2020). Further permission was sought and approved by the Ministry of Health and Social Services Ethics Committee (Ref: 17/3/3MA). This study followed the ethical principles of the Belmont Report through the application of respect for persons, beneficence, and justice. The study’s purpose and objectives were discussed with each participant, including the participants’ rights to voluntary participation and withdrawal without any consequences. For participants in the age groups 0-10 and 11-20 years, written informed consent was obtained from their parents or legal guardians. Those who agreed to participate in the study provided written informed consent. Participants’ data were anonymised during data collection to ensure confidentiality and were encrypted and stored in a password-protected laptop. Furthermore, all methods were carried out under relevant guidelines and regulations.

## Results

### Quantitative results

A total of 4477 ETR.net records were reviewed, among which 2846 (63.6%) were from the Oshana region and 1631 (36.4%) were from the Kunene region. From the data analysed, the following were the outcomes as categorised into completed, cured, defaulted, died and failed: 58.36%, 33.02%, 1.92%, 6.32%, 0.37% for Kunene, and 34.76%, 48.57%, 4.91%, 9.76%, 1.99% for Oshana region, respectively.

The mean age was 37.2 ± 18.3 and 36.5 ± 18.7 in the Oshana and Kunene regions. The demographic characteristics are summarised in Table 1.

**Table 1:** Age and sex demographics of TB records in the Kunene and Oshana regions

		**Number of TB records per region (%)**	
**Age groups**		**Kunene**	**Oshana**
0–10		148 (9.1)	195 (6.9)
11–20		143 (8.8)	183 (6.4)
21–30		335 (20.5)	672 (23.6)
31–40		384 (23.5)	760 (26.7)
41–50		274 (16.8)	479 (16.8)
51–60		161 (9.9)	254 (8.9)
61 +		186 (11.4)	303 (10.6)
Total		1631 (100)	2846 (100)
Mean ± SD		36.5 ± 18.7	37.2 ± 18.3
Minimum		0	0
Maximum		108	104
Sex	Male	927 (56.8)	1707 (60.0)
	Female	704 (43.2)	1139 (40.0)
Type of patient	Newly registered	1462(89.6)	2627(92.3)
	Transferred in	169(10.4)	219(7.7)
Type of Tuberculosis	Pulmonary	1341(82.2)	2149(75.5)
	Extrapulmonary	290(17.8)	697(24.5)
Type of DOT	Guardian (relative, neighbour)	35(4.7)	1913(70.2)
	Workplace	3(0.4)	11(0.4)
	Health Facility	221(29.6)	704(25.8)
	Community-based	488(65.3)	99(3.6)

Table 1 above summarises the demographic variables from the regions. There were a total of 1631 and 2846 cases in the Kunene and Oshana regions, respectively. In the Kunene region, the oldest case was 108 years old, while 104 was the age of the oldest case in the Oshana region. The mean ± SD was nearly identical in both regions, at 36.5 ± 18.7 and 37.2 ± 18.3 in the Kunene and Oshana regions, respectively. In both regions, more than half the population was comprised of male cases. At least 88% of the cases in both regions were newly registered, the remainder having had transferred in from other regions. In terms of the type of TB, pulmonary TB cases in the Kunene and Oshana regions were 82.2% and 75.5%, respectively. Extrapulmonary TB was 17.8% and 24.5% in the Kunene and Oshana regions, individually. The majority of the cases in the Kunene and Oshana regions utilised community-based DOTS (65.3%) and guardian DOTS (70.2%).

The overall aggregated (from 2016–2020) TB-TO for Kunene and Oshana regions revealed that only 50.6% and 43.9% of the TB cases were successfully treated, respectively. They are summarised in Table 2. Pearson Chi-squared cross-tabulations revealed a positive, albeit negligible, association between the Kunene region’s treatment outcome and DOT type (*p* = 0.006). Contrastingly, no association was observed between the Oshana region’s TB-TO and age group (*p* = 0.066) (Table 2).

**Table 2:** TB treatment outcomes (TB-TO) and success rate in Kunene and Oshana regions

Treatment outcome	Kunene		Oshana	
Completed	383(36.3)		559(34.8)	
Cured	533(32.7)		781(48.6)	
Defaulted	31(2.9)		79(4.9)	
Died	102(6.3)		157(9.8)	
Failed	6(0.4)		32(2.0)	
Treatment Success Rate (with P-value)				
Successful	826 (50.6)	0.006*	1248 (43.9)	0.066
Unsuccessful	805 (49.4)		1598 (56.1)	

^*^: *p*-value =  < 0.05

Table 3 indicates that in the Oshana region, guardian DOT was the main contributor to both treated (71.3%) and untreated (69.2%) cases. Moreover, in the Kunene region, the main contributor to both treated (60.8%) and untreated (72.6%) cases was community-based DOTS played a major role in among the untreated TB cases. However, as indicated in Table 2 above, it is clear that as a statistically significant contributor to poor TB-TOs in the Kunene region, this variable was central to the questions asked in the interviews with key informants.

**Table 3:** Type of DOT and treatment outcomes

TREATMENT OUTCOME	Kunene				Oshana			
	**Type of DOT**				**Type of DOT**			
	**Guardian**	**Workplace**	**Health Facility**	**Community-Based**	**Guardian**	**Workplace**	**Health Facility**	**Community-Based**
**Treated**	5.6%	0.6%	32.9%	60.8%	71.3%	0.6%	25.7%	2.4%
**Not treated**	3.2%	0.0%	24.2%	72.6%	69.2%	0.3%	25.9%	4.7%

The logistic regression analysis results in Table 4 between TB-TO and the several independent variables revealed that in the Kunene region, only the Type of DOT used (Community-based DOTS with adjusted odds ratio [aOR] = 0.356, 95% Confidence Interval [CI] = 0.835–2.768, *p* = 0.006) was found to have a statistically significant association with TB-TO. Meanwhile, in the Oshana region, only the Age Group variable had a significant effect on TB-TO. Specifically, age groups 21–30 years old (aOR = 1.643, 95% CI = 1.005–2.686, *p* = 0.048), 31–40 years old (aOR = 1.725, 95% CI = 11.026–2.9, *p* = 0.040), 41–50 years old (aOR = 2.003, 95% CI = 1.155–3.476, *p* = 0.013) and 51–60 years old (aOR = 2.106, 95% CI = 1.228–3.612, *p* = 0.007) had statistically significant associations with poor TB-TO.

**Table 4:** Adjusted odds ratios for treatment outcomes estimated by logistic regression

Region	Variable	Successful treatment	Un successful treatment	*P*-value	aOR (95% CI)
	Type of DOT				
Kunene	Guardian	26(5.60%)	9(3.20%)	0.675	
	Workplace	3(0.60%)	0(0.00%)	0.243	0.765(0.218–2.680)
	Health Facility	152(32.90%)	69(24.20%)	0.154	2.028(0.619–6.6430)
	Community-based	281(60.80%)	207(72.6%)	0.006*	0.356(0.835–2.768)
	HIV status				
	Negative	357(73.50%)	366(72.20%)		
	Positive	129(26.50%)	137(27.00%)	0.299	1.303(0.790–2.149)
	Unknown	0(0.00%)	4(0.80%)		
	Age group				
	0–10 years old	74(9.00%)	74(9.20%)	0.842	1.104 (0.417–2.923)
	11–20 years old	82(9.910%)	61(7.60%)	0.433	0.713(0.306–1.662)
	21–30 years old	189(22.90%)	146(18.10%)	0.442	0.717(0.307–1.674)
	31–40 years old	191(23.10%)	193(24.00%)	0.271	1.641(0.679–3.966)
	41–50 years old	129(15.60%)	145(18.00%)	0.723	0.836(0.309–2.257)
	51–60 years old	79(9.60%)	82(10.20%)	0.560	1.324(0.515–3.406)
	+ 61 years old	82(9.90%)	104(12.90%)		
	Sex				
	Male	473(57.3%)	454(56.40%)		
	Female	353(42.7%)	351(43.60%)	0.492	1.157(0.763–1.756)
	Bacteriological result				
	Negative	98(20.10%)	84(16.60%)		
	Positive	365(74.90%)	333(65.70%)	0.354	0.78(0.461–1.319)
	Not Tested	7(1.40%)	56(11.00%)	0.051	13.038(1.475–115.271)
	Smear not done	17(3.50%)	34(6.70%)	0.560	1.337(0.503–3.558)
Oshana	Type of DOT				
	Guardian	873(71.3)	1040(69.2)	0.208	
	Workplace	7(0.6)	4(0.3)	0.320	0.532 (0.153–1.849)
	Health Facility	315(25.7)	389(25.9)	0.667	0.948 (0.742–1.21)
	Community-based	29(2.4)	70(4.7)	0.073	1.916 (0.942–3.896)
	HIV Status				
	Negative	516(66.4)	552(61.3)	0.540	1.073 (0.857–1.342)
	Positive	260(33.5)	374(38.5)	0.871	1.26 (0.078–20.345)
	Unknown	1(0.1)	2(0.2)		
	Age group				
	0–10 years old	91(7.3)	104(6.5)	0.177	1.493 (0.834–2.673)
	11–20 years old	85(6.8)	98(6.1)	0.294	1.296 (0.799–2.103)
	21–30 years old	323(25.9)	349(21.8)	0.048*	1.643 (1.005–2.686)
	31–40 years old	323(25.9)	437(27.3)	0.040*	1.725 (1.026–2.9)
	41–50 years old	205(16.4)	274(17.1)	0.013*	2.003 (1.155–3.476)
	51–60 years old	107(8.6)	147(9.2)	0.007*	2.106 (1.228–3.612)
	+ 61 years old	114(9.1)	189(11.8)		
	Sex				
	Male	737(59.1)	970(60.7)		
	Female	511(40.9)	628(39.3)	0.55	1.065 (0.865–1.312)
	Bacteriological result				
	Negative	56(7.2)	72(7.8)	0.303	0.813 (0.548–1.206)
	Positive	522(67.0)	571(62.1)	0.208	1.332 (0.853–2.08)
	Not Tested	101(13.0)	167(18.2)	0.164	0.716 (0.446–1.147)
	Smear not done	100(12.8)	109(11.9)		

^*^: *p*-value =  < 0.05

### Qualitative results

The researcher collected data from the six and seven key informants from Kunene and Oshana regions, respectively. Their baseline characteristics are presented in Table 5 below:

**Table 5:** Baseline characteristics of key informants in Kunene and Oshana regions

Characteristic	Kunene		Oshana		
Sex	Male	Female	Male	Female	
	2	4	2	6	
Health Facility	District Hospital		Intermediate Hospital	Health Centre	Clinic
	6		2	3	3

Table 5 above provides baseline characteristics of key informants in both the Kunene and Oshana region. There were two males and four females in the Kunene region, making a total of six interviewees. All worked at the three District Hospitals, each hospital providing two participants. In the Oshana region, there were two males and six females, making up for a total of eight participants. Two participants worked at the intermediate hospital, three worked at the health centres, and the last three worked at the clinics.

Inductive thematic analysis identified two themes and two sub-themes for the Kunene region, while the Oshana region had two themes and three sub-themes (as shown in Table 6 below).

**Table 6:** Themes and sub-themes identified for Kunene and Oshana regions

Region	Themes	Sub-themes
Kunene	Geographical access	Transport unavailability and Vastness of the area
	Access to healthcare services	Nomadism
Oshana	Substance Abuse	Alcoholism and tobacco smoking
	Adherence	Stigma
		Health education

#### Kunene region’s theme 1: Geographical access

The inability to reach and observe patients taking their medication may be a fundamental impediment to the foundation of the DOTS program. Under this theme, the following sub-themes were generated. Examples of the participants’ verbatim comments are presented in italics.

#### Sub-theme 1: Transport unavailability and vastness of the area

It was revealed that the lack of transport within the regions was one of the significant impediments to either healthcare workers directly observing patients or the patients' access to DOTS services. One participant said, “*Challenges include no transport… collecting sputum and seeing if a patient is taking medicine is difficult*.” P#4 Agreeing with the first respondent, another participant said, “*Transport challenges to go out and trace… and give education… and then the staff is delayed until transport is available*.” P#5 One participant identified distance as a barrier to accessing DOTS services, saying, “*Opuwo is vast… some patients stay 180 km away from the hospital. It is tough to reach them for treatment.*” P#3.

#### Kunene region’s theme 2: Access to Healthcare Services

Nomadism is a specific cultural pattern that influences health-seeking behaviour and access to healthcare services. This means that nomadic patients initiated on TB treatment may miss out on such services over time, owing to their culture.

#### Sub-theme 1: Nomadism

Probing revealed that several TB patients in the Kunene region were nomads, and it proved difficult to access them once treatment was initiated. Some of the participants responded as follows: “… *sometimes you don’t know where they are, they change their addresses… to find them is just very difficult. P#1.*” and “*Patients in Opuwo are very nomadic… they don’t have cell phones to help find them. P#5* Two others reported that: *“…some move to Angola after treatment initiation, and we don’t see them again…” P#6* and “… *Number two, is that the patients are nomadic… the patients are moving up and down.”* P#4.

#### Oshana region’s theme 1: Substance abuse

Alcohol and tobacco consumption while on TB treatment may have unfavourable effects on treating the disease. The following sub-theme was identified.

#### Sub-theme 1: Alcoholism and tobacco smoking

Several participants reported that alcohol abuse in the adult age range in the region was common. One participant said: “*We experience a lot of alcohol and medicine mixing… It's mostly not the young patients. It’s mostly the people between 30–50 years there, that’s where the problem is… mainly the men because they drink more and smoke tobacco they don’t mention it to us, but that patient’s guardian informs us to ask them about their alcohol use”* P#12 Another one reported that “*In some cases, you can have a patient coming to take treatment here as DOT… then after some months coming for a sputum smear test, the result will come again positive… if you ask further he will even tell you “Yeah I drink a little katombo* [home brewed alcohol] *or beer just a little…” that way medication can be interfered with by alcohol*.” P#8.

#### Oshana region’s theme 2: Adherence

Continued probing revealed non-adherence to TB treatment in the Oshana region among adult patients. Below are the identified sub-themes.

#### Sub-theme 1: Stigma

Stigma can negatively affect adherence to treatment among TB patients. One participant respondent said, “*You know TB came a long, long time ago… and people were just kind of isolated… If you get TB, you start sleeping alone; you use your cup or whatsoever. That thing is still in the community… Because if you tell the family that this person is now on TB treatment when you do the follow-up, you will find that this person has been stigmatised… And after the person was stigmatised, they will start to stigmatise themselves, saying don’t touch my cup or things I now have TB.*” P#7 Another participant revealed that: “*Some patients feel they have ideas of stigmatisation. They think if they come here every day… the other patients will see them and maybe they will label them that one is suffering from TB disease. Mostly grown men are afraid of this.*” P#8 This can be attributed to male patients having different health-seeking behaviour as they avoid going to hospitals to prevent the feeling of being stigmatised.

#### Sub-theme 2: Health education

The lack of proper TB understanding and management among patients can also affect treatment outcomes. It was revealed that adult male patients might be complicit with this sub-theme. One responded said: “*Sometimes patients do not follow health education. Some they listen, some don’t – normally they are men… they are difficult.”* P#11 Participants also suggested understanding the health education given to patients is paramount in improving patient adherence, particularly among those who transferred in from other regions.

## Discussion

This study aimed to identify, analyse and describe the factors related to the unsuccessful treatment outcomes in Oshana and Kunene regions. The quantitative analysis revealed that in the Kunene region, only the Type of DOT had a significant association with TB-TO. Meanwhile, in the Oshana region, only the Age Group variable was significantly associated with poor TB-TO. The qualitative data analysis revealed that Kunene faces TB control challenges due to geographical access to health care services and nomadism, while Oshana struggles with substance abuse and adherence issues related to stigma and health education. In this study, TB was higher among male patients. This is consistent with findings from a 2016 systematic review suggesting that men are less likely to seek out TB management in numerous situations in low to middle-income nations [13]. Sex was found to be insignificant between treatment outcomes in both regions. Despite this finding, qualitative results indicate that male TB patients in the Oshana region had poor health-seeking behaviour. This was observed in several African studies, suggesting that females have better health-seeking behaviours and favourable treatment outcomes [14–16]. In the current study, TB prevalence was highest among the age groups of 21–40, with Kunene and Oshana regions recording 44% and 50.3%, respectively, possibly due to the increased social habits of patients in this age range [17].

Qualitative data corroborates this finding, with respondents indicating that middle-aged patients are the most affected age group. Respondents suggested that treatment failure in the Oshana region is primarily due to alcohol and tobacco use during TB treatment among patients within this age group. This coincides with a previous study that reported that middle-aged patients were prone to alcohol and drug abuse—effectively adversely affecting the treatment of the disease [18]. Kibuule et al. [9] explained that adults in similar age groups, from lower to middle-income countries, are predisposed to poor TB-TOs, such as loss to follow-up, as they are a high-risk group for unemployment, HIV, alcohol and drug misuse, smoking, and multiple sexual partners.

The reported relationship between substance abuse and the age group (21–40) in this study’s qualitative results could have caused poor adherence to anti-TB medication, negatively affecting TB-TO. Furthermore, poor adherence could have also resulted in patients not receiving adequate health education about TB and its treatment to change their attitudes towards the illness. Other reasons for poor adherence may include “forgetting to take medication, being away from home, drug side effects, being unable to go to the health facilities on the date of appointment and being hospitalised” [19]^p.10^. Ultimately, poor treatment adherence can result in treatment failure, drug resistance and death [20, 21].

Directly Observed Therapy Short course is a WHO-approved strategy for increasing commitment toward the management of TB by requiring health professionals, community volunteers, or family members to watch and monitor patients receiving each dosage [22]. It typically includes community-based DOTS (CB-DOTS) and facility-based DOTS (FB-DOTS), among others. This study points to the Kunene region’s heavy reliance on CB-DOTS for TB treatment, accounting for most treated cases. Consistent with other studies, CB-DOTS outperformed different types of DOTS regarding positive treatment outcomes [9, 23, 24]. A probable reason for this could be that within the Kunene region, the community health workers were well-embedded within the Ministry of Health and Social Services’ community activities, providing the necessary treatment services and support, despite the challenges of distance travelled to observe patients and the patients’ nomadic culture. In a comparison with the entire Namibian TB-TO, annual treatment success rates significantly increased when FB-DOTS was expanded to CB-DOTS [25]. However, as a predictor of unsuccessful treatment outcomes, the results also indicate that CB-DOTS accounted for 72.6% of all untreated cases. Commonly, nomadic populations are challenged with accessing health care, leading to increased morbidity and mortality [26]. Ultimately, this can contribute to the underperformance of the region’s TSR in the Kunene region.

To meet the WHO objective of 'End TB' by 2035, CB-DOTS should be improved by curating new strategies specific to the environment to identify patients at risk of poor treatment outcomes [25]. Even though CB-DOTS accounted for the majority of all the successfully and unsuccessfully treated cases in the Kunene region, more attention is needed to mitigate the challenges being faced by patients and healthcare workers to improve the region’s TB-TO.

The multiple regression analysis did not reveal an association between DOTS treatment outcomes and sex, bacteriological results, or HIV status. Multiple studies have found the characteristics mentioned above to be significant predictors of DOTS treatment outcomes [14, 27–29]. Further studies could explore how specific types of DOTS in the Kunene region may result in better TB-TO using stepwise regression methods. Knowledge gaps remain regarding how TB treatment outcomes differ by age in the Oshana region and countries with high TB/HIV co-infection.

Lastly, the Oshana region had a better TB-TO when compared to the Kunene region, although none had met the TSR indicator. Predominantly urban areas seemed to have better TB-TO and are at higher odds of successful TB-TO among patients when compared to rural areas [30, 31]. Similar findings were made in this study when a better TB-TO was recorded in the Oshana region, a peri-urban area, while Kunene is primarily rural. This may be due to adequate patient follow-up systems in urban hospitals and increased access to health services [32, 33].

## Conclusions

TB treatment outcomes varied by the type of DOT and age group in the Kunene and Oshana regions, respectively. Rigorous community health education about TB treatment and risk factors is crucial in improving adherence to TB treatment. These findings may be used in implementing TB control interventions in peri-urban and rural contexts in Namibia. Moreover, it is essential to improve the robustness of surveillance systems used in both regions to improve regional treatment success rates.

### Limitations

In this study, only the sociodemographic variables collected from ETR.net were included. Additionally, listwise deletion was used to account for missing variables and may introduce bias and limit the generalisability of findings. Moreover, the study excluded the insights of the patients on TB treatment during the qualitative data collection phase. All participants were, however, encouraged to be honest in their responses.

## Data Availability

The data supporting the current study’s findings are available from the corresponding author upon reasonable request.
